# Estimated Travel-Related CO_2_ Emissions Avoided Through Virtual Care for Inflammatory Bowel Disease Patients in Remote Regions of Ontario, Canada

**DOI:** 10.3390/healthcare14152425

**Published:** 2026-08-06

**Authors:** Luke J. Nguyen, Vivian W. Huang, Peter Habashi, Parul Tandon

**Affiliations:** 1 Mount Sinai Centre for Inflammatory Bowel Disease, Toronto, ON M5G 1X5, Canada; luke.nguyen@sinaihealth.ca (L.J.N.); vivian.huang@sinaihealth.ca (V.W.H.);; 2 Faculty of Health Sciences, Queen’s University, Kingston, ON K7L 3N6, Canada; 3 Division of Gastroenterology and Hepatology, University Health Network and Sinai Health System, University of Toronto, Toronto, ON M5G 2C4, Canada; 4 Institute of Health Policy, Management and Evaluation, University of Toronto, Toronto, ON M5T 3M6, Canada; 5 ICES, Toronto, ON M4N 3M5, Canada

**Keywords:** inflammatory bowel diseases, telemedicine, carbon emissions, greenhouse gases, sustainable development, rural health services, health equity, Ontario

## Abstract

**Highlights:**

**What are the main findings?**

- A virtual IBD care program for patients living at least 100 km from a tertiary centre was estimated to avoid approximately 487 metric tonnes of travel-related CO_2_ emissions under the base-case assumption across 2222 visits over eight years.
- Each virtual visit was estimated to have replaced an average of 829 km of round-trip travel, corresponding to approximately 219 kg of CO_2_ avoided per encounter in the base-case scenario.

**What are the implications of the main findings?**

- Virtual care can deliver a meaningful estimated reduction in travel-related emissions for geographically disadvantaged chronic-disease populations.
- Embedding virtual visits in routine IBD management offers health systems a practical lever to advance both equitable access and decarbonization goals simultaneously, although prospective evaluation of clinical outcomes, patient satisfaction, and induced demand is required.

**Abstract:**

**Background/Objectives:** Inflammatory bowel disease (IBD) requires frequent specialist follow-up, yet patients in remote areas face significant geographic barriers to care. Healthcare delivery also contributes substantially to greenhouse gas (GHG) emissions, with patient travel a major source. The Promoting Access and Care through Centres of Excellence (PACE) virtual care program was designed to improve access for IBD patients in underserved regions of Ontario. This study estimated travel-related carbon dioxide (CO_2_) emissions potentially avoided. **Methods:** A retrospective cohort study was conducted using the PACE registry at the Mount Sinai Hospital IBD Centre between June 2016 and October 2024. Adults with Crohn’s disease, ulcerative colitis, or IBD-unclassified residing ≥100 km from the Centre and completing ≥1 virtual visit were included. Two-way travel distance avoided was calculated using Google Maps, adjusting for any travel to local facilities. Carbon emissions were estimated using a composite base-case factor of 260 g CO_2_/km with predefined sensitivity analyses covering alternative vehicle-mix and transport scenarios. **Results:** Among 389 IBD patients, 2222 virtual visits were completed. The mean (±SD) two-way distance avoided per visit was 843 ± 812 km, corresponding to 219 ± 211 kg CO_2_ per visit. The mean cumulative reduction per patient was 1252 ± 2245 kg CO_2_, totalling 487,104 kg (487 tonnes) over eight years. **Conclusions:** The PACE program was associated with substantial estimated reductions in travel-related CO_2_ emissions for IBD patients in remote Ontario, with results robust across vehicle mix and transport assumptions. Virtual care may offer a scalable pathway advancing both equitable access and healthcare-sector decarbonization.

## 1. Introduction

Inflammatory bowel disease (IBD), comprising Crohn’s disease, ulcerative colitis, and IBD-unclassified, is a chronic relapsing condition that requires lifelong specialist management. Patients often experience unpredictable flares and complications necessitating close follow-up. For individuals in rural or remote regions, access to subspecialty care is constrained by distance and concentration of tertiary centres, which contributes to disparities in access, higher costs, and delays in treatment [1].

The COVID-19 pandemic accelerated the adoption of virtual care technologies across most specialty disciplines, prompting a rapid system-wide shift in service delivery that would otherwise have taken years [2]. This transition prompted concurrent interest in the environmental implications of health service delivery, particularly the potential co-benefits of reduced patient travel. Post-pandemic, virtual care has remained embedded in many chronic-disease programs, providing an opportunity to evaluate whether observed environmental effects persist beyond the acute-response period.

Healthcare delivery also carries significant environmental consequences. The Canadian healthcare system generates substantial greenhouse gas (GHG) emissions through its operations and supply chains, and globally health systems account for an estimated 4–5% of GHG output [3,4]. Frameworks such as the National Health Service (NHS) England Delivering a “Net Zero” National Health Service strategy provide methodological guidance for healthcare-sector carbon accounting and identify patient travel as a substantial and modifiable component of the sector’s footprint [5,6]. Travel for health visits accounts for 10% of overall healthcare emissions [6], making reduced travel an attractive target for sustainability.

Virtual care offers a strategy to improve access while reducing environmental impact. Life cycle analyses demonstrate that telemedicine lowers per-visit emissions compared with in-person encounters, primarily by avoiding travel. Thiel et al. reported that virtual visits significantly reduced greenhouse gas emissions per patient [7]. Systematic reviews have confirmed that telemedicine consistently decreases travel-related carbon dioxide emissions across diverse clinical settings [8,9]. Specialty studies have supported these findings. For example, Morcillo-Serra et al. documented measurable reductions in emissions from digital consultations in Spain [10]. More recently, Marković et al. reported reductions in travel distance and travel-related CO_2_ emissions associated with telehealth consultations in an IBD cohort in Southeast Europe, providing initial disease-specific evidence [11]. These results suggest that virtual care can enhance access while contributing to environmental sustainability, although program-specific and long-term estimates in the IBD population remain limited.

The PACE telemedicine program was launched at the Mount Sinai Hospital IBD Centre in 2016 to address geographic disparities in access to subspecialist IBD care in Ontario. The program’s original rationale was to reduce patient travel burden, shorten wait times for specialist consultations, and support equitable care in rural and remote communities [12]. Because the program has operated continuously across the pre-pandemic, pandemic, and post-pandemic periods, it provides a naturalistic setting in which to estimate the environmental correlates of a structured virtual-care initiative in a chronic-disease population.

Despite these observations, long-term, program-specific environmental estimates for structured virtual IBD care remain scarce. The majority of prior analyses have been cross-sectional or centred on pandemic-era rapid expansion [7,8,13]. The objective of the present analysis was to estimate travel-related CO_2_ emissions potentially avoided through the PACE virtual IBD program among patients residing at least 100 km from the tertiary centre, applying a composite emission factor with predefined sensitivity analyses that reflect evolving Canadian vehicle fleet composition and transportation patterns.

## 2. Materials and Methods

### 2.1. Study Design and Population

A retrospective cohort study was conducted of patients enrolled in the Promoting Access and Care through Centres of Excellence (PACE) virtual care program at the Mount Sinai Hospital Inflammatory Bowel Disease (IBD) Centre, Toronto, Canada. Eligible participants were identified from the PACE registry and included adults with a confirmed diagnosis of Crohn’s disease, ulcerative colitis, or IBD-unclassified who resided at least 100 km from the IBD Centre and who participated in at least one virtual visit between 1 June 2016 and 30 October 2024. A residence threshold of 100 km one-way from the tertiary centre was pre-specified as part of the PACE program’s eligibility criteria, informed by prior Canadian rural health services research, and was selected to identify patients for whom travel to the Centre represented a substantial burden and a meaningful potential source of transport-related emissions [12]. Because distance eligibility was a condition of enrolment in the PACE program itself, all patients in the PACE registry during the study window met the inclusion criteria and were included in the analysis; no patients were excluded.

### 2.2. Data Sources

Demographic and clinical data were extracted from the PACE registry. Information collected included age, sex, and IBD diagnosis. The registry prospectively recorded the number and dates of virtual visits during the study period. A single, most recent residential address on record for each patient was used to calculate travel distances across all of that patient’s visits. Because address history was not available in the registry, changes of residence during the eight-year observation period could not be accounted for.

### 2.3. Estimation of Travel Distance Avoided

For each participant, the two-way travel distance between the participant’s most recent residential address on record and the Mount Sinai Hospital IBD Centre was calculated using Google Maps (www.google.com/maps, accessed between October and December 2024, Google LLC, Mountain View, CA, USA). Queries were performed between October and December 2024, using the shortest available driving route on the then-current road network; where multiple routes were suggested, the default (fastest) route was selected. If a patient was required to travel to a local facility to access the virtual visit, the two-way distance to that facility was subtracted from the total distance avoided. The cumulative distance avoided was then calculated for each participant by summing all avoided round trips across the observation period.

### 2.4. Estimation of Carbon Emissions Avoided

Carbon dioxide emissions potentially avoided in the base-case analysis were estimated using the approach described by Galts et al., who derived vehicle-related emission factors from U.S. Environmental Protection Agency data (approximately 249 g CO_2_ per kilometre for an average-sized passenger vehicle) [14]. To account for non-tailpipe emissions and road life-cycle impacts, a 10% adjustment was applied, while a 5% reduction was incorporated to reflect the proportion of Canadians who rely on public transit or use electric vehicles [15]. The resulting composite emission factor was 260 g CO_2_ per kilometre travelled. Emissions associated with video-based virtual appointments were considered negligible, consistent with prior analyses estimating less than 0.03 kg CO_2_ per visit (<0.01% of an in-person encounter) [7]. Although Galts et al. excluded appointments exceeding 200 km round-trip on the assumption that longer trips were less likely to be undertaken entirely by private vehicle, the present analysis retained all eligible visits within the primary analysis because many patients from remote Ontario communities routinely travel long distances by private vehicle, and additional transport-mode uncertainty was addressed through sensitivity analyses (see Section 2.5). The emissions avoided per patient were calculated as the product of distance avoided and the emission factor. Cumulative reductions per participant and total emissions avoided across the cohort were determined.

The system boundary for this analysis was restricted to travel-related tailpipe and life-cycle CO_2_ emissions associated with return trips between the patient’s residence and the Mount Sinai Hospital IBD Centre for scheduled outpatient clinic visits. Emissions arising from digital infrastructure, video-conferencing platforms, patient-side computing devices, and operation of local facilities used to access virtual encounters were excluded from the primary calculation. Prior life-cycle analyses have suggested that these components contribute a small fraction of total per-visit emissions [7,16].

### 2.5. Sensitivity Analysis

To characterize uncertainty in the primary estimate, three predefined sensitivity scenarios were constructed, varying assumptions about vehicle mix and transportation mode. In the conservative scenario, all travel was assumed to be undertaken by an average internal combustion engine (ICE) passenger vehicle, applying an unadjusted factor of approximately 274 g CO_2_/km (representing the Galts et al. [14] factor of 249 g/km with the 10% road life-cycle adjustment retained, and no offset for EV or transit share). In the electric vehicle (EV) scenario, the Ontario zero-emission vehicle (ZEV) share of new motor vehicle registrations was estimated from Statistics Canada data as the mean of the shares at the start and end of the study period (0.78% in the first quarter of 2017 and 9.34% in the fourth quarter of 2024), giving an average of approximately 5.1% [17]. A life-cycle emission factor of 52 g CO_2_e/km for battery electric vehicles was applied, corresponding to the International Council on Clean Transportation estimate for vehicles charged on a low-carbon electricity grid [18]. Applying this EV factor to the estimated ZEV share and the ICE factor to the remaining share gave an effective composite factor of approximately 263 g CO_2_/km. In the higher-adoption scenario, EV share was set to 15% and rail travel to 5% (rail factor approximately 35 g CO_2_e/passenger-km for national rail [19]), with the remainder assumed to be ICE, giving an effective composite factor of approximately 229 g CO_2_/km. For each scenario, effective emission factors were applied to the total travel distance avoided to derive scenario-specific estimated cumulative emissions.

### 2.6. Statistical Analysis

Mapping of the geographic areas serviced by the PACE program was performed in R (version 4.5.1; R Foundation for Statistical Computing, Vienna, Austria) using the *ggplot2*, *ggspatial*, and *maps* packages. Descriptive statistics were used to summarize demographic and clinical characteristics of the cohort. Continuous variables were reported as mean ± standard deviation (SD) or median with interquartile range (IQR) where appropriate, while categorical variables were presented as frequencies with percentages as n/N (%). Per-visit statistics were calculated across all 2222 virtual visits, with each participant contributing one observation per visit. Per-participant statistics were calculated across the 389 participants. The primary outcomes were mean travel distance avoided per participant and mean CO_2_ emissions avoided per participant and per visit. Total cohort-level reductions in travel distance and emissions were also reported. Statistical analyses were conducted using IBM SPSS Statistics for Windows, Version 29.0 (IBM Corp., Armonk, NY, USA).

### 2.7. Ethics

The study was conducted in accordance with the Declaration of Helsinki and approved by the Sinai Health System Research Ethics Board (REB# 10-0112-E; initially approved 13 July 2016). Although the present analysis was retrospective, written informed consent for the prospective collection of clinical data into the PACE registry was obtained from all participants at the time of enrolment.

## 3. Results

**Figure 1:**
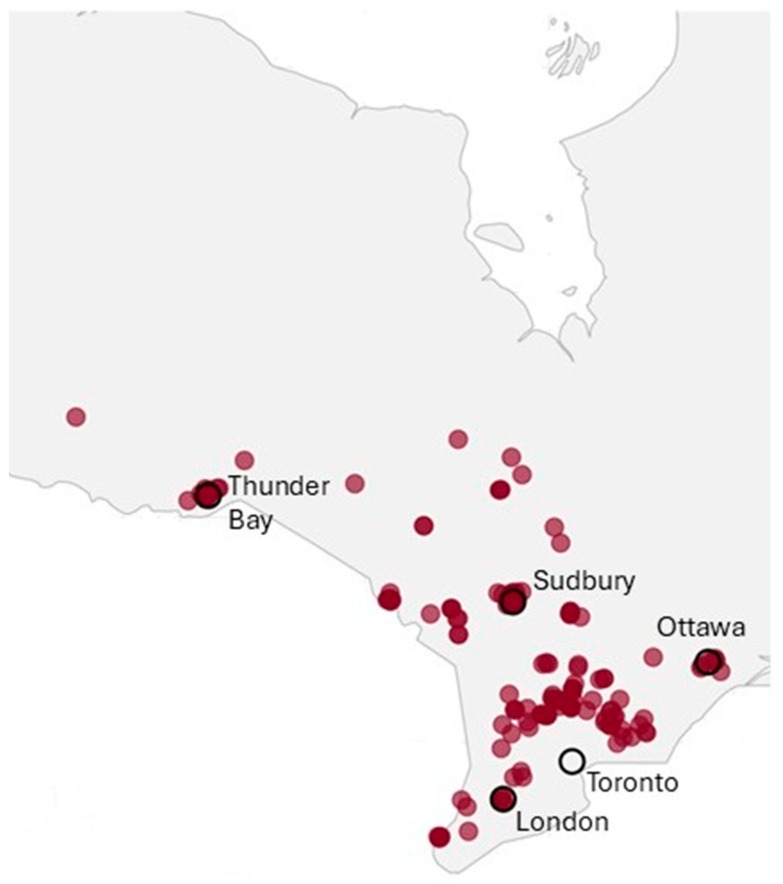
Geographic mapping of locations of participants in the PACE virtual IBD program. Burgundy circles indicate locations, and black hollow circles mark major Ontario cities (Toronto, Ottawa, London, Sudbury, and Thunder Bay) for geographic reference.

A total of 389 patients meeting eligibility criteria (residence ≥ 100 km from the Centre and ≥1 completed virtual visit) were identified in the PACE registry between June 2016 and October 2024. As distance eligibility was a condition of PACE enrolment, this represented all patients in the registry during the study window, with no exclusions. Of these, 233/389 (60%) had Crohn’s disease, 128/389 (33%) had ulcerative colitis, and 28/389 (7%) had IBD-unclassified. The cohort demonstrated a female predominance (241/389, 62%) and was composed predominantly of younger adults, with 206/389 (53%) between 25 and 44 years of age, 132/389 (34%) between 45 and 64 years of age, and 51/389 (13%) 65 years and older. In total, patients completed 2222 virtual video visits over the 8-year period (mean 5.7 ± 5.6 visits per patient). Figure 1 shows the geographic coverage of virtual care services provided by the PACE program.

**Figure 2:**
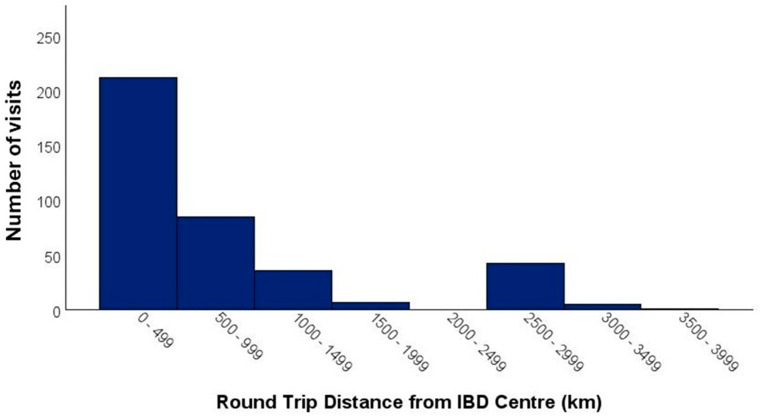
Distribution of virtual visit round-trip distances avoided, showing most virtual encounters replaced travel under 500 km, with fewer long-distance visits exceeding 2500 km.

The distribution of round-trip travel distance avoided per specialist visit was bimodal (Figure 2). The mean (±SD) two-way travel distance avoided per virtual visit was 843 ± 812 km, while the median (interquartile range, IQR) was 472 (624) km. More than half of participants (213/389, 55%) avoided travel between 200 and 500 km per visit, whereas 49/389 (12.6%) avoided more than 2500 km of round-trip travel. Over the study period, virtual visits avoided a total of 1,873,476 km of travel. The mean cumulative travel distance avoided per participant was 4816 ± 8636 km, while the median (IQR) was 2130 (4506) km.

Assuming private automobile travel and a carbon emission factor of 260 g CO_2_/km (base-case scenario), the mean (±SD) and median (IQR) reduction in emissions per visit were 219 ± 211 kg CO_2_ and 123 (162) kg CO_2_, respectively. The mean (±SD) and median (IQR) cumulative reduction per participant were 1252 ± 2245 kg CO_2_ and 554 (1172) kg, respectively. Aggregated across all visits, virtual encounters were estimated to have prevented approximately 487,104 kg CO_2_ (487 metric tonnes) under the base-case scenario.

Estimated total CO_2_ emissions avoided varied predictably across the three sensitivity scenarios (Table 1). In the conservative all-ICE scenario (emission factor 274 g CO_2_/km), the estimated total was approximately 513,333 kg CO_2_ (513 metric tonnes). In the EV scenario, applying a life-cycle EV emission factor to the estimated Ontario ZEV share (effective factor 263 g CO_2_/km), the estimated total was approximately 492,724 kg CO_2_ (493 metric tonnes), closely matching the base-case estimate. In the higher-adoption scenario incorporating 15% EV share and 5% rail travel (effective factor 229 g CO_2_/km), the estimated total was approximately 429,026 kg CO_2_ (429 metric tonnes). The direction and rough magnitude of the estimated environmental benefit were preserved across all scenarios.

## 4. Discussion

The present analysis provides estimates of travel-related CO_2_ emissions potentially avoided through virtual gastroenterology care delivered via the PACE program in remote regions of Ontario. Among 389 patients completing 2222 virtual visits over eight years, the estimated cumulative reduction in the base-case scenario was approximately 487 metric tonnes of CO_2_, with sensitivity scenarios yielding totals between approximately 429 and 513 metric tonnes. These figures should be interpreted as model-based estimates rather than directly measured environmental effects, given the absence of a contemporaneous in-person comparison group and the reliance on assumed vehicle and mode mixes.

The consistency of the estimated benefit across sensitivity scenarios suggests that virtual care delivery for geographically dispersed chronic-disease populations may contribute meaningfully to healthcare-sector decarbonization objectives, even under conservative assumptions about vehicle fleet composition and transport-mode substitution.

The current findings extend prior research on the environmental correlates of virtual care while differing methodologically in important respects. Habashi et al. described the PACE telemedicine initiative as a means of improving equity of access for IBD patients in remote communities, noting marked reductions in travel burden and wait times for specialist consultation [12]. Building on this foundation, the current analysis quantifies the environmental benefit of that model. Welk et al. estimated system-wide travel and emission reductions during pandemic-era virtual-care expansion in Ontario using administrative data across specialties [13], whereas the present analysis examines a single, structured, IBD-focused program operating continuously across pre-pandemic, pandemic, and post-pandemic periods. Marković et al. estimated travel-related emissions avoided through telehealth in an IBD cohort in Southeast Europe [11], applying different distance calculations and emission factors reflecting local vehicle fleet and transit landscape. Direct numerical comparison is therefore limited, but the direction of effect is consistent. Galts et al. applied similar U.S. EPA-derived emission factors to Canadian gastroenterology clinic visits [14] but excluded appointments exceeding 200 km round-trip on the assumption that longer trips were less likely to be undertaken entirely by private vehicle. The present study retained long-distance visits in the primary analysis, recognizing that patients from remote Ontario communities may indeed travel long distances by private vehicle. The higher-adoption sensitivity scenario partially addresses residual uncertainty about mode substitution on very long trips.

Interpretation of the estimated emission reduction rests on the assumption that each virtual encounter replaced a face-to-face visit that would otherwise have occurred at the same frequency. In practice, low-friction virtual care may enable induced demand. For example, there may have been additional brief check-ins that would not have been scheduled in a purely in-person model. These induced visits would partially offset the estimated per-visit savings, although the direction of the net aggregate effect was not quantified. Whether such induced demand generates net clinical value beyond the environmental question is beyond the scope of the current analysis but warrants prospective evaluation, particularly given the potential for improved disease control and reduced acute care utilization in chronic-disease cohorts.

The cohort was restricted to patients who completed at least one virtual visit, thereby excluding remote patients who were unable to access virtual care because of connectivity, device, or language barriers, or who disengaged from tertiary follow-up altogether. The included cohort, therefore, likely represents a relatively engaged and resourced subset of the remote IBD population. The extrapolated per-capita environmental estimate may not fully generalize to the broader remote population. Complementary approaches such as mobile healthcare units with intermediate depots have been proposed as alternative strategies to reach unmet-need populations in geographically dispersed settings [20].

The observation window (June 2016 to October 2024) spans pre-pandemic, pandemic, and post-pandemic periods, during which virtual care availability, patient acceptance, transportation patterns, and Canadian vehicle fleet composition changed considerably. Annual visit volumes were not analyzed, and a formal temporal stratified analysis by pre-pandemic, pandemic, and post-pandemic periods was not performed. No inference is therefore drawn about how visit volumes, or the associated emission estimates, varied across these periods. This represents a limitation of the current work.

Beyond environmental correlates, these findings have implications for health policy and health equity. Patients with IBD living in rural or northern communities often face geographic barriers to care that exacerbate disease outcomes and healthcare costs. Virtual gastroenterology visits are now endorsed by Choosing Wisely Canada for clinically appropriate scenarios and represent a potential strategy to bridge these gaps while contributing to national climate targets within the healthcare sector [21]. Zurl et al. reported that patient travel accounts for a substantial proportion of healthcare-related carbon emissions globally, suggesting that even modest reductions in visit-related travel could translate to meaningful environmental gains at the system level [22]. Integrating carbon accounting into models of care could further support sustainable decision-making in healthcare resource allocation, a growing policy priority within both the Canadian Institute for Health Information (CIHI) and the Canadian Association of Gastroenterology’s emerging climate-health initiatives.

This study’s contributions to the literature are threefold. First, it provides one of the first long-term model-based estimates of travel-related carbon emissions avoided in a single, structured specialty care program in Canada. Second, it shows that a structured virtual IBD care program has been continuously delivered over an eight-year period spanning pre-pandemic, pandemic, and post-pandemic phases, providing a naturalistic setting for evaluating environmental correlates of virtual care beyond the acute pandemic response. Third, it introduces an evaluative framework with sensitivity analyses that can be adapted to other chronic disease programs to estimate carbon avoidance through telehealth implementation.

There are additional limitations that merit consideration. First, all reported values are model-based estimates rather than directly measured environmental effects. Second, although sensitivity analyses were conducted using alternative emission factors reflecting Ontario EV adoption over the study period and modest rail substitution, individual-level data on vehicle type, fuel, carpooling, and mode of transport were not available. Actual emissions may vary in either direction from the modelled scenarios. Additionally, a single most recent residential address was applied retrospectively to all of each patient’s visits, as address history was not available. Any changes of residence over the eight-year period were therefore not captured and may have led to over- or under-estimation of individual travel distances. The direction of any resulting bias is not systematic. Moreover, the primary analysis retained visits exceeding 2500 km of round-trip travel, some of which may in reality have involved a combination of car, coach, and short-haul flight segments. This uncertainty is only partially addressed by the higher-adoption sensitivity scenario. The system boundary excluded emissions associated with digital infrastructure, patient-side devices, and operation of local facilities used to access virtual encounters. Prior life-cycle analyses have, however, suggested these components contribute a small fraction of total per-visit emissions [7,16]. The analysis did not assess clinical outcomes, patient satisfaction, disease control, acute care utilization, or health equity outcomes. Finally, as a single-program single-centre analysis, generalizability may be limited to comparable healthcare settings with similar geography, infrastructure, and vehicle fleet.

Future directions include integrating prospective data collection on both clinical and environmental outcomes, developing emission dashboards within telehealth platforms, and evaluating hybrid care models that optimize patient access while minimizing environmental costs. Multi-centre collaborations across Canadian IBD centres could provide standardized national estimates of healthcare-related carbon emissions, including formal stratified temporal analyses by pre-pandemic, pandemic, and post-pandemic phases, supporting the development of sustainability metrics for future health policy planning.

## 5. Conclusions

In summary, this analysis provides model-based estimates suggesting that virtual gastroenterology care delivered through the PACE program was associated with substantial reductions in travel-related CO_2_ emissions among IBD patients residing in remote regions of Ontario, with sensitivity scenarios yielding total estimated reductions between approximately 429 and 513 metric tonnes over eight years. These findings support the further evaluation of structured virtual care as one component of climate-conscious and equitable chronic-disease service delivery, while highlighting the need for prospective, multi-centre studies incorporating clinical outcomes, patient-reported measures, and standardized carbon accounting to move from estimated to directly measured environmental effects.

## Figures and Tables

**Table 1:** Sensitivity analyses of estimated total CO_2_ emissions avoided under alternative vehicle-mix and transportation assumptions.

Scenario	Assumed Vehicle/Mode Mix	Effective Emission Factor (g CO_2_/km)	Estimated Total CO_2_ Avoided
Base case (Galts et al. approach; 5% EV/transit reduction)	95% private ICE + 5% EV/transit reduction (per [14,15])	260	487,104 kg (487 t)
Conservative (all-ICE)	100% private ICE, no EV or transit offset	274	513,333 kg (513 t)
EV scenario (Ontario ZEV share)	≈5.1% Ontario EV share (mean of 2017 and 2024); ~95% ICE; life-cycle EV factor 52 g/km	263	492,724 kg (493 t)
Higher adoption (15% EV + 5% rail)	15% EV, 5% national rail, 80% ICE	229	429,026 kg (429 t)

ICE, internal combustion engine; EV, electric vehicle; t, metric tonnes. Total travel distance avoided (1,873,476 km) was multiplied by each scenario’s effective emission factor to derive scenario-specific totals.

## Data Availability

The data supporting the findings of this study are held in the PACE registry at the Mount Sinai Hospital IBD Centre. Restrictions apply to the availability of these data, which were used under institutional data governance for the current study and are not publicly available. Aggregated, de-identified data may be made available from the corresponding author upon reasonable request and with permission of the institution.
